# Retention and Predictors of Attrition Among People Living With HIV on Antiretroviral Therapy in Guinea: A 13-Year Historical Cohort Study in Nine Large-Volume Sites

**DOI:** 10.3389/ijph.2023.1605929

**Published:** 2023-07-13

**Authors:** Niouma Nestor Leno, Foromo Guilavogui, Alioune Camara, Kadio Jean-Jacques Olivier Kadio, Timothé Guilavogui, Thierno Saidou Diallo, Mamadou Aliou Diallo, Daniel William Athanase Leno, Button Ricarte, Youssouf Koita, Laye Kaba, Arnold Ahiatsi, Nagnouman Touré, Pascal Traoré, Souleymane Chaloub, André Kamano, Carlos Arias Vicente, Alexandre Delamou, Mohamed Cissé

**Affiliations:** ^1^ African Center of Excellence for Prevention and Control of Communicable Diseases (CEA-PCMT), Faculty of Health Sciences and Techniques, Gamal Abdel Nasser University, Conakry, Guinea; ^2^ Department of Public Health, Faculty of Health Sciences and Techniques, Gamal Abdel Nasser University Conakry, Conakry, Guinea; ^3^ Ministry of Health, Conakry, Guinea; ^4^ National AIDS and Hepatitis Control Program, Conakry, Guinea; ^5^ National Malaria Control Program, Conakry, Guinea; ^6^ Center of Infectious Disease Research and Training, Gamal Abdel Nasser University of Conakry, Conakry, Guinea; ^7^ Gynaecology-Obstetrics Service, Donka National Hospital, Conakry, Guinea; ^8^ Merck Group, Lyon, France; ^9^ NGO “Doctors Without Borders Belgium”, Conakry, Guinea; ^10^ National Center for Education and Research in Rural Health Maférinyah, Forécariah, Guinea; ^11^ Department of Dermatology and Sexually Transmitted Infections, Gamal Abdel Nasser University of Conakry Faculty of Health Sciences and Techniques, Conakry, Guinea

**Keywords:** predictors, antiretroviral therapy, HIV/AIDS, retention, attrition

## Abstract

**Objectives:** The objective of this study was to estimate the retention rate of patients in an ART program and identify the predictors of attrition.

**Methods:** This was a historical cohort study of HIV patients who started ART between September 2007 and April 2020, and were followed up on for at least 6 months in nine large-volume sites. Kaplan Meier techniques were used to estimate cumulative retention and attrition probabilities. Cox proportional hazards models were used to identify predictors of attrition.

**Results:** The cumulative probability of retention at 12 and 24 months was 76.2% and 70.2%, respectively. The attrition rate after a median follow-up time of 3.1 years was 35.2%, or an incidence of 11.4 per 100 person-years. Having initiated ART between 2012 and 2015; unmarried status; having initiated ART with CD4 count <100 cells/μL; and having initiated ART at an advanced clinical stage were factors significantly associated with attrition.

**Conclusion:** The retention rate in our study is much lower than the proposed national target (90%). Studies to understand the reasons for loss to follow-up are needed.

## Introduction

The scale of the HIV epidemic has changed dramatically over the past 30 years. Once a fatal disease, HIV infection is now considered as a chronic and manageable disease [1]. This transformation is mainly due to advances in antiretroviral therapy (ART), which has substantially reduced AIDS incidence and related morbidity and mortality [1, 2]. The number of AIDS-related deaths has been reduced by more than 60% globally since its peak in 2004. Also, new infections in children have decreased by 52% between 2010 and 2019 [3].

For this reason, global initiatives promoted by UNAIDS (ending the HIV epidemic by 2030) and the World Health Organization (Test and Treat) [4, 5], have been set up to improve access to antiretroviral therapy for people living with HIV. As a result, by the end of June 2020, 26 million people were on antiretroviral therapy worldwide [3].

However, patient retention in care remains a challenge, particularly in sub-Saharan African countries [6–8] and undermines efforts to improve treatment outcomes [6]. The results of a meta-analysis of 32 studies of ART programs in sub-Saharan Africa showed that only 80% of patients who initiated ART were still on treatment after 1 year, 77% after 2 years, and 72% after 3 years. Loss of follow-up and death were the main causes of patient attrition from the antiretroviral therapy program [6], and it is seen as a threat to the sustainability of HIV treatment programs [8]. It has been shown that this fallout from care is a serious form of resource waste [6].

With a national HIV prevalence of 1.5%, Guinea has a generalized HIV epidemic. After the introduction of free ART in 2007, several changes were made to the HIV management protocol in Guinea. ART initiation with a CD4 count less than 200 cells/μL between 2007 and 2012; ART initiation with a CD4 count less than 350 cells/μL between 2012 and 2015; ART initiation with a CD4 count less than 500 cells/μL between 2015 and 2017; introduction of the “Treat All” strategy (putting HIV-positive people on ART regardless of their CD4 count) between 2017–2020 [10, 11]. Despite this commitment, access to antiretroviral treatment and the quality of care for people living with HIV are still low in Guinea (insufficient diagnosis and management of opportunistic infections, poor food and nutrition management, etc.) [9]. In December 2021, only 52% of the 120,000 people living with HIV in Guinea had access to antiretroviral treatment [10].

Also, previous studies have shown that low retention of HIV-infected patients on treatment remains a serious problem in antiretroviral treatment programs in Guinea. Thus, according to a study, the rate of loss to follow-up among patients who initiated antiretroviral therapy between 1st May 2017 and 30th June 2019 was 26.0% [11]. Another study realized in 2016 showed low retention of patients in the ART program at 12 months after initiation (78.7%) in Guinea [12]. In recent years, the Guinean government, in collaboration with its technical and financial partners, has invested enough in public health interventions to improve retention of patients on ART and to increase ART coverage. At the time of review and planning, it is essential to evaluate key interventions of the AIDS and hepatitis program in Guinea. It is from this perspective that the present study was envisaged. The objective of this study was to estimate the retention rate of patients in the ART program in Guinea and identify the predictors or factors associated with attrition (no retention).

## Methods

### Study Design

We conducted a historical cohort study by using routine individual follow-up data of patients who initiated antiretroviral therapy in Guinea between September 2007 and April 2020.

### Study Setting

This study was conducted in high-volume care sites for people living with HIV (PLHIV) in Guinea. In the Guinean context, a site is considered high-volume when the number of people living with HIV it serves is 250 or more. In 2019, 29 out of 142 sites nationally met this definition, and covered more than 90% of all patients on ART in the country. The large-volume sites are present in each of the eight Guinean health regions [13]. This study focused on nine large-volume sites (eight in Conakry and one in Nzérékoré) that have electronic databases allowing automatic and rapid extraction of information on the long-term follow-up of HIV patients. The study sites were chosen on the basis of the availability of electronic databases that could allow for analysis of retention over a long period of follow-up on ART. Because of the poor quality of completion and archiving of primary follow-up data management tools for patients living with HIV, sites without an electronic longitudinal follow-up database were not included in this study. The total number of active patients on ART followed in these nine sites was 24,682 [14], or 40% of the national cohort [15].

### Study Population

The study population consisted of children under 15 years of age (9–14 years) and adults 15 years and older who were confirmed HIV-positive and started ART during the period of 1 September 2007 to 30 April 2020, with a minimum of 6 months of follow-up prior to the data extraction date. The data extraction date was 31 October 2020. The length of follow-up in this study was chosen to address the need for information in Guinea regarding long-term retention of patients on ART (retention by age groups, sex, and time to follow-up). Patients who had not started triple antiretroviral therapy during the study period and patients for whom data needed to calculate follow-up time were missing were excluded.

### Sample Size and Sampling Criteria

Participants were selected from the registration database of patients who started on antiretroviral therapy at the study centers. We performed an exhaustive sampling of patients who met the inclusion criteria for this study. A total of 23,686 patients were obtained for the different analyses.

### Study Variables

Variables extracted for the analysis of retention and attrition included socio-demographic characteristics, clinical and therapeutic characteristics, biological markers (CD4), the ART initiation year, and patient monitoring status (lost to follow-up, deceased, transferred, and attrition) (Table 1).

**TABLE 1:** Measurement of outcomes in the follow-up of 23,686 HIV-infected patients who started antiretroviral therapy between 2007 and April 2020 in 9 high-volume care sites (Conakry and Nzérékoré, Guinea. 2021).

Outcome	Operational definition
Lost to follow-up	A patient was considered lost to follow-up if he/she did not come to the treatment site for at least 3 months (90 days) after the date of last appointment for clinical visit, and for whom no information on health status was known before the date of data extraction. This definition was chosen to align with national guidelines for monitoring patients on ART in Guinea [9]. Patients lost to follow-up had the event on the date of the last clinic visit
Deceased patient	Deceased patients had the event of interest on the date of death, if not available, on the date of last visit to the site
Transferred out patient	Any patient referred to another service providing ART before the date of data extraction was considered transferred. These patients were censored on the date they were transferred
Attrition	The proportion of patients who have interrupted or stopped ART because they are considered lost to follow-up or deceased during the study. For the attrition rate estimation, uncensored patients were considered “patients with attrition,” i.e., not retained in the ART program; and censored patients were considered “patients without attrition,” i.e., retained in the ART program. A patient was classified as censored if he or she had a formally registered transfer to another site or was still being followed at the site at the end of the study period, i.e., at the date of data extraction

### Data Collection

The data for this study were extracted from two databases: TIER.NET (managed by NGO Doctors Without Borders Belgium) and AVICEMA (managed by Mission PhilAfricaine), then exported to Excel for processing and cleaning. To ensure confidentiality, information on patients’ surnames, first names, affiliation, physical address, and contacts (telephone number and e-mail address) were excluded when extracting data from the different study sites. Only the patient code, also known as the unique identification number assigned by the sites following the instructions of the national aids and hepatitis program, was used to identify the patients included in this study.

### Data Analysis

The data was analyzed using SPSS version 21 software. The primary outcome was attrition, defined as death and loss to follow-up. Kaplan Meier techniques (life tables and Kaplan Meier curves) were used to estimate the probabilities of retention and attrition of patients in the ART care program throughout the follow-up timeline. In the univariate analysis, Kaplan Meier curves were plotted to assess retention according to potential risk factors, and the Mantel Haenszel log-rank test was used to compare survival curves. The choice of variables included in the Kaplan Meier curves and in the Cox proportional regression model was guided by the results of the scientific literature relating to the factors associated with attrition or non-retention. We estimated the association between predictor variables and attrition among patients on ART using a univariate Cox proportional hazards regression model. The crude relative risk or hazard ratio (HR) and its 95% confidence interval was used as the measure of association.

The identification of factors that predicted attrition was performed using a multivariable Cox proportional hazard model. This allowed us to calculate the adjusted hazard ratios and their 95% confidence intervals. The proportional hazards assumption was evaluated using Kaplan-Meier plots against Cox predictions and the results of these analyses suggested that the proportional hazards assumption is valid.

To identify independent associated factors of attrition, first variables were selected into the multivariable model based using a stepwise forward strategy (*p*-value < 0.1). Due to their clinical and biological relevance in the retention of HIV patients in the ART program, we ensured that the variables “sex and level of care” were among the initial variables selected. Second, stepwise backward elimination was used until all remaining variables were significantly associated with attrition (*p*-value < 0.05).

## Results

### Enrolment of Participants

**FIGURE 1:**
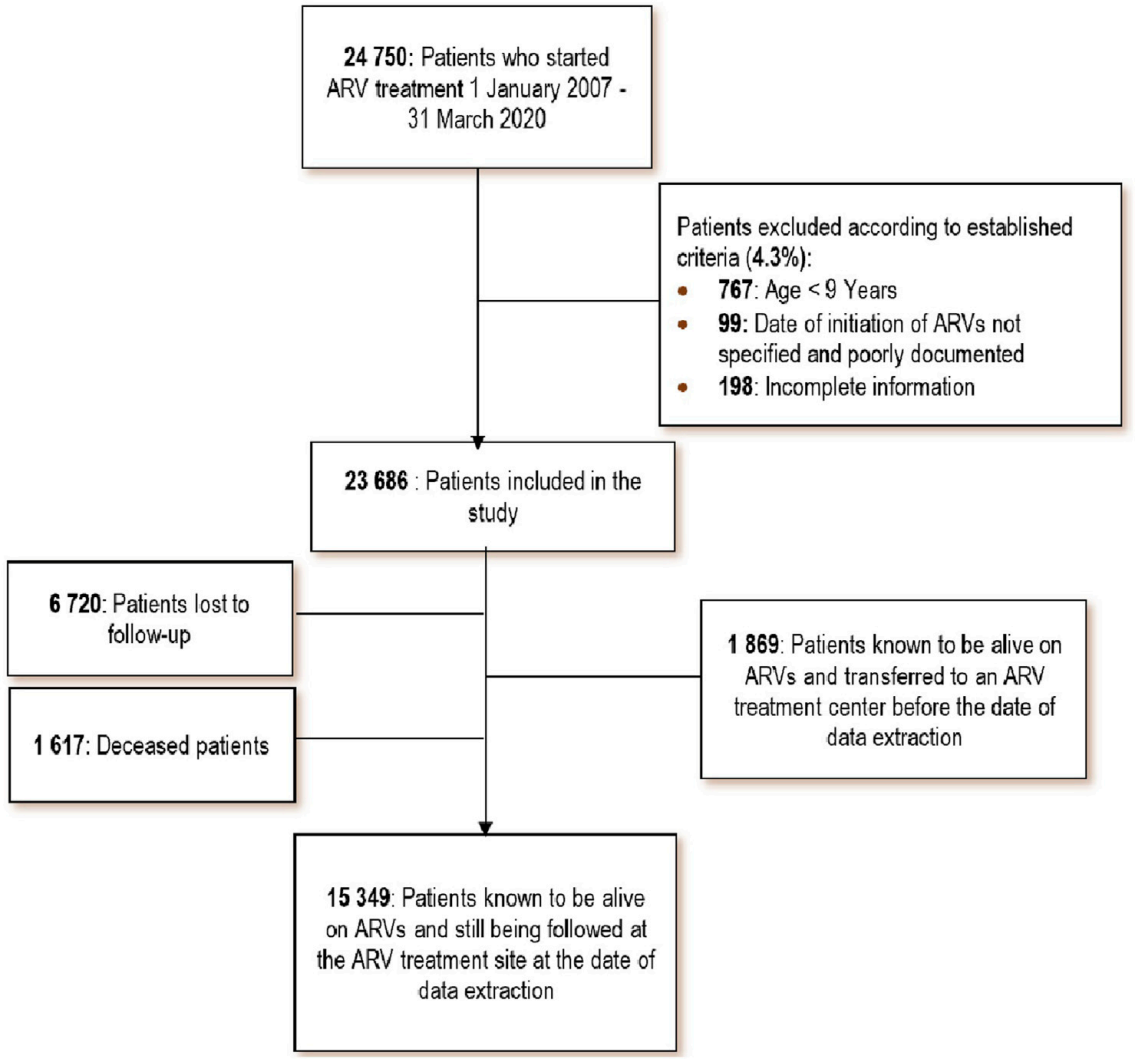
Flow of patients enrolled in the study (Conakry and Nzérékoré, Guinea. 2021).

A total of 24,750 people living with HIV were enrolled for antiretroviral treatment between September 2007 and April 2020 in the nine sites involved in this study. After excluding 1,064 patients who did not meet the eligibility criteria, a total of 23,686 patients were included in the analysis (Figure 1).

### Characteristics of Participants

The median follow-up time for all 23,686 patients was 37.2 months (IQR: 15.7–71.6), or 3.1 years. The median age was 37 years (IQR: 30–46). The majority of patients were women (69.0%). Nearly half (47.7%) had initiated ART at an advanced clinical stage of HIV infection, or stages III and IV of the WHO classification. The majority (40.8%) of patients in this study had initiated ART during the 2017–2020 period, which corresponds to the implementation period of the “Treat All” approach in the ART program in Guinea. Patients who initiated ART between 2012 and 2015 (the peak period of the Ebola epidemic in Guinea) represented 17.0% of the sample (Table 2).

**TABLE 2:** Baseline characteristics of 23,686 HIV-infected patients who started antiretroviral therapy between 2007 and April 2020 at 9 high-volume care sites (Conakry and Nzérékoré, Guinea. 2021).

Characteristics	N	(%)
Total	23,686	100.0
Site area		
Conakry	20,787	87.8
Interior	2,899	12.2
Year of initiation of ART		
2007–2012	4,684	19.8
2012–2015	4,023	17.0
2015–2017	5,319	22.5
2017–2020	9,660	40.8
Legal status of the site		
Private	2,899	12.2
Public	20,787	87.8
Site level in the pyramid of care		
Level 3	2,899	12.2
Level 2	8,456	35.7
Level 1	12,331	52.1
Sex		
Women	16,345	69.0
Man	7,341	31.0
Age Median (IQR)	37 (30–46)	
Age group		
Adults (15 years and older)	23,371	98.7
Children (under 15)	315	1.3
Marital status		
Married	18,072	76.3
Not married	5,606	23.7
Missing	8	0.0
Employment status		
Employee	8,912	37.6
Unemployed	14,764	62.3
Missing	10	0.0
Type of HIV		
HIV type 1	23,534	99.4
Other HIV types (Type 2 or Type 1 + 2)	132	0.6
Missing	20	0.1
CD4 count in cells/μL, Median (IQR) (*n = 23,641*)	250 (212–500)	
Category of CD4 count in cells/μL		
>350	7,703	32.5
200–350	7,766	32.8
100–200	4,168	17.6
<100	4,004	16.9
Missing	45	0.2
WHO Stage of HIV infection		
Early stage (I and II)	12,381	52.3
Advanced stage (III and IV)	11,305	47.7
ART regimens		
TDF + 3TC + EFV	18,855	79.6
AZT + 3TC + NVP	2,284	9.6
Other ART regimes of first line	2,547	10.8

ART, antiretroviral therapy; IQR, interquartile range; n, Number of observations; WHO, World health organization; Level 1: Health center and private practice, Level 2: Regional or Prefectural hospital and private clinic that has the rank of a regional or prefectural hospital, Level 3: National hospital or private clinic that has the rank of a national hospital.

### Retention in the Antiretroviral Therapy Program

The 37.2 months or 3.1 years of follow-up time of the 23,686 patients who started ART between September 2007 and April 2020 represents 73,427 person-years of follow-up. At the end of the study period, 1,617 (6.8%) had died, and 6,720 (28.4%) were lost to follow-up; refer to Figure 1.

The cumulative probability of retention was 64.8% (95% CI: 64.2%–65.4%). This cumulative probability at 12, 24, 48, 120, and 156 months was 76.2%; 70.2%; 64.0%; 53.6%, and 48.0%, respectively. The probability of retention for women at 12 months (78.7%; CI95%: 78.2%–78.9%) was better than for men (73.1%; CI95%: 72.61%–73.79%) (Table 3).

**TABLE 3:** Cumulative probability of retention in the ART program throughout the follow-up period by sex of 23,686 HIV-infected patients who initiated antiretroviral therapy between September 2007 and April 2020 in 9 large-volume care sites (Conakry and Nzérékoré, Guinea. 2021).

Follow-up time	Total		Women		Man	
	Number of patients at risk	Percent (95% CI)	Number of patients at risk	Percent (95% CI)	Number of patients at risk	Percent (95% CI)
6 months	22,566	86.0 (85.1–86.8)	15,595	87.6 (87.2–87.8)	6,971	84.3 (84.03–84.76)
1 year	19,093	76.2 (76.1–77.2)	13,305	78.7 (78.2–78.9)	5,789	73.1 (72.61–73.79)
2 years	14,244	70.2 (69.3–71.6)	10,067	73.2 (72.6–73.5)	4,177	67.1 (66.52–67.62)
3 years	11,578	66.5 (65.7–68.2)	8,221	70.2 (69.5–70.3)	3,357	64.0 (63.52–64.69)
4 years	9,108	64.0 (62.7–65.6)	6,511	67.6 (65.8–67.8)	2,597	61.4 (60.7–62.23)
5 years	7,113	61.3 (60.4–62.9)	5,121	65.2 (63.9–65.7)	1,993	58.5 (58.01–59.5)
6 years	5,691	58.4 (57.9–61.4)	4,127	62.7 (62.1–63.7)	1,565	55.4 (55.1–57.2)
7 years	4,714	55.8 (58.9–59.7)	3,401	61.6 (60.8–61.9)	1,313	54.4 (53.6–55.2)
8 years	3,828	54.7 (53.1–56.7)	2,760	60.2 (58.5–62.3)	1,068	52.7 (52.0–53.8)
9 years	2,906	54.4 (53.6–55.9)	2,079	58.1 (57.4–58.7)	828	50.8 (48.9–52.9)
10 years	2,315	53.6 (53.1–54.5)	1,668	57.1 (55.8–56.7)	648	48.9 (48.5–50.4)
11 years	1,769	53.0 (52.2–53.4)	1,275	55.7 (54.2–55.3)	6,971	48.2 (47.3–49.2)
12 years	965	50.0 (49.8–51.5)	685	54.1 (53.2–54.5)	5,789	46.1 (45.0–47.8)
13 years	194	48.0 (47.1–50.9)	136	53.5 (52.4–53.9)	4,177	42.9 (41.5–45.6)

CI, confidence interval.

**FIGURE 2:**
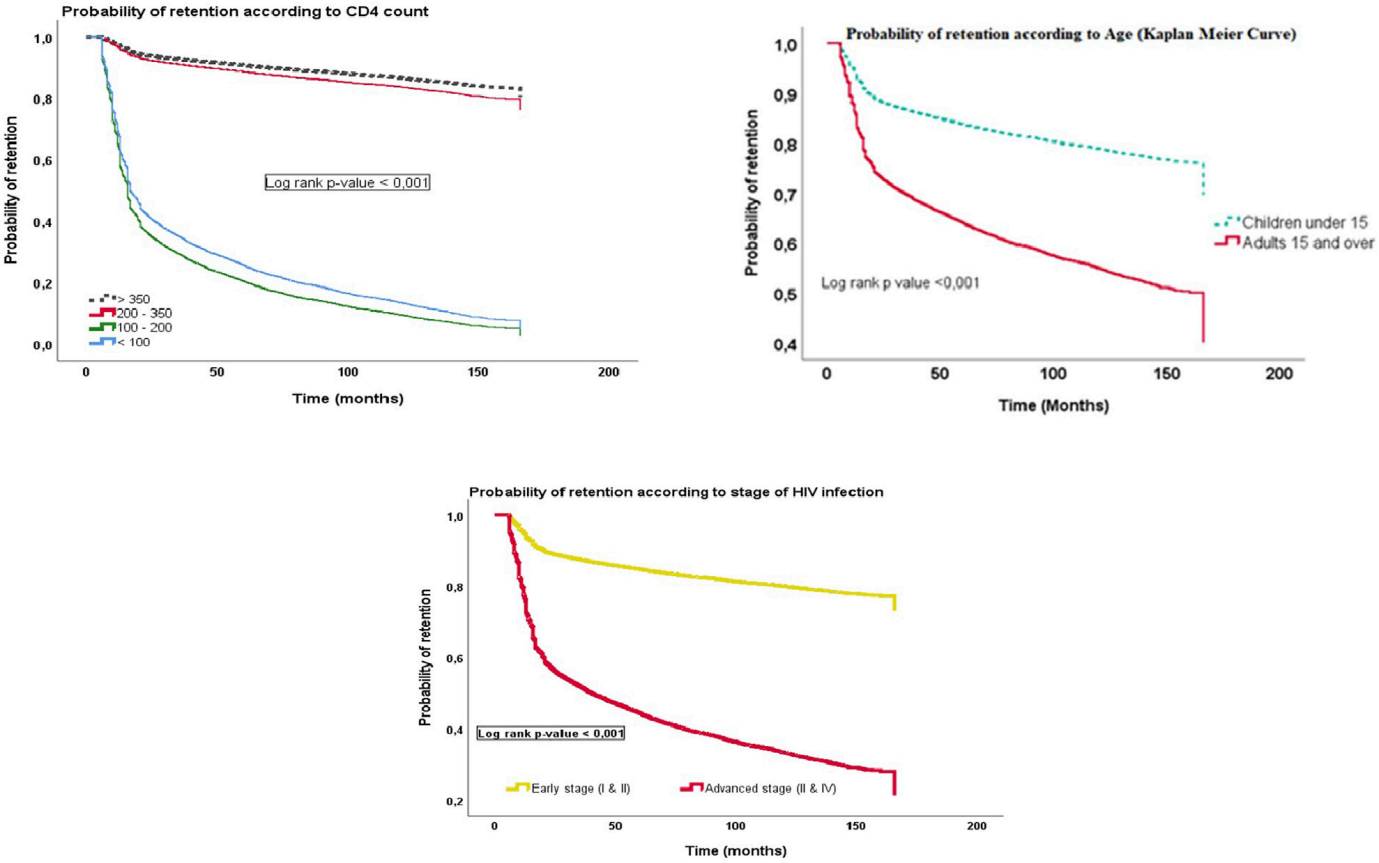
Probability of retention over time according to sociodemographic, clinical, and biological characteristics of 23,686 HIV-infected patients who started antiretroviral therapy between September 2007 and April 2020 in 9 care sites (Conakry and Nzérékoré, Guinea. 2021).

We also found that the 12-month retention probability for children under 15 years of age (92.5%; 95% CI: 90.9%–94.0%) was better than for adults 15 years and older (76.5%; 95% CI: 76.3%–76.8%). The Kaplan Meier retention curves for patients who started antiretroviral therapy with high CD4 counts were higher in trend than those for patients with low CD4 counts. Similarly, the Kaplan Meier curve for the probability of retention of patients who started antiretroviral therapy at an early clinical stage (I and II of the WHO classification) of HIV infection had a higher trend than that of patients who started therapy at an advanced clinical stage (III and IV). The differences observed between the Kaplan Meier curves on the probability of retention were significant, with a log-rank test *p*-value < 0.001 (Figure 2).

### Trends in the Components of Attrition

Of the 23,686 patients included in the study, 8,337 were patients no longer retained in the antiretroviral therapy program, for a cumulative probability of attrition of 35.2% (95% CI: 32.2%–37.2%), representing a cumulative incidence of attrition of 11.4 cases per 100 person-years. Of the 8,337 who experienced attrition, 1,618 (19.4%) were deceased and 6,719 (80.5%) were lost to follow-up. If all patients included in the study are considered, loss to follow-up accounted for 28.4% and deaths accounted for 6.8% of the sample at the end of the study. Table 4 shows that the probability of attrition increases as a function of the length of follow-up of patients receiving ART. This increased from 14.0% at 6 months to 23.8% at 12 months of follow-up to 46.4% at 120 months (10 years) and reached 52.0% at 156 months (13 years). This upward trend over time is also observed for lost to follow-up status and for deceased status.

**TABLE 4:** Cumulative probability of death, of being lost to follow-up, and of attrition of 23,686 patients infected with HIV and having initiated antiretroviral therapy between September 2007 and April 2020 in 9 large-volume care sites (Conakry and Nzérékoré, Guinea. 2021).

Follow-up time	Number of patients at risk	Cumulative incidence (95% CI)		
		Death	Lost to follow-up	Attrition
6 months	22,566	2.4% (2.1–2.5)	11.6% (11.3–11.8)	14.0% (13.5–14.78)
1 year	19,093	4.2% (4.0–4.4)	19.6% (19.4–20.1.)	23.8% (23.1–24.2)
2 years	14,244	5.6% (5.5–5.9)	24.2% (23.3–24.6)	29.8% (28.9–30.2)
3 years	11,578	6.8% (6.6–7.2)	26.7% (26.5–27.3)	33.5% (32.3–34.8)
4 years	9,108	7.6% (7.5–8.6)	28.4% (28.2–29.3)	36.0% (34.5–37.9)
5 years	7,113	9.4% (9.1–9.6)	29.3% (29.10–31.2)	38.7% (36.8–39.1)
6 years	5,691	10.2% (9.9–10.5)	31.4% (30.8–31.8)	41.6% (38.9–41.9)
7 years	4,714	10.4% (10.1–10.6)	33.8% (33.3–34.3)	44.2% (40.7–44.9)
8 years	3,828	10.7% (10.5–11.4)	34.6% (34.2–35.8)	45.3% (42.5–46.1)
9 years	2,906	10.9% (10.8–11.2)	34.7% (34.6–36.1)	45.6% (43.5–45.9)
10 years	2,315	11.5% (11.3–11.7)	34.9% (33.0–36.7)	46.4% (45.2–46.9)
11 years	1,769	12.0% (11.6–12.1)	35.0% (34.5–39.8)	47.0% (46.6–47.6)
12 years	965	12.2% (12.0–12.9)	37.8% (37.2–41.6)	50.0% (48.5–50.3)
13 years	194	12.7% (12.6–14.3)	39.3% (38.9–41.1)	52.0% (49.5–52.5)

CI, confidence interval.

### Predictors of Attrition

At multivariate analysis, having initiated ART between 2012–2015 vs. between 2017–2020 (aHR 2.81: 2.172–3.643); initiated ART as an adult (15 years and older) vs. as a young person (younger than 15 years) (aHR 1.50: 1.102–2.002); unmarried status vs. married status (aHR 1.182: 1.013–1.347); initiated ART with CD4 count <100 cells/μL vs. with CD4 count > 350 cells/μL (aHR 5.92: 5.091–6.543); having initiated ART at an advanced clinical stage (stages III and IV WHO classification) vs. at an early stage (stage I and II WHO classification) (aHR 3.66: 3.045–3.975) were factors significantly associated with attrition in this study (Table 5).

**TABLE 5:** Univariate and multivariate analysis of the predictors of attrition among 23,686 patients infected with HIV and having initiated antiretroviral therapy between September 2007 and April 2020 in 9 large-volume care sites (Conakry and Nzérékoré, Guinea. 2021).

Characteristics	Total	Person year	Attrition^$^	Attrition (%)	Attrition (1,000 person-years)	Univariate analysis			Multivariate analysis		
						Crude HR	*p*-value	95% CI	aHR	*p*-value	95% CI
Total	23,686	73,427	8,337	35.2	11.4						
Site area											
Conakry	20,787	64,440	7,426	35.7	11.5	1.10	0.008	(1.075–1.186)	NS	NS	
Interior	2,899	8,987	911	31.4	10.1	1					
Year of initiation of ART											
2007–2012	4,684	14,520	1,550	33.1	10.67	1.12	0.070	(0.981–1.321)	1.09	0.065	(0.764–1.26)
2012–2015	4,023	11,471	1,835	45.6	15.99	2.78	<0.001	(2.096–3.564)	2.81	<0.001	(2.172–3.643)
2015–2017	5,319	16,489	1,809	34.0	10.97	1.23	<0.001	(1.120–1.961)	1.27	<0.001	(1.131–2.102)
2017–2020	9,660	29,946	3,002	31.1	10.02	1			1		
Legal status of the site											
Private	2,899	8,987	911	31.4	10.1	1					
Public	20,787	64,440	7,426	35.7	11.5	1.12	0.008	(1.027–1.186)	NS	NS	
Site level in the pyramid of care											
Level 3	2,899	8,987	911	31.4	10.1	1					
Level 2	8,456	26,214	3,233	38.2	12.3	1.19	<0.001	(1.080–1.281)			
Level 1	12,331	38,226	4,193	34.0	10.9	1.06	0.236	(0.875–1.132)	NS	NS	
Sex											
Women	16,345	50,670	5,463	33.4	10.8	1					
Man	7,341	22,757	2,874	39.1	12.6	1.25	<0.001	(1.186–1.301)	NS	NS	
Age group											
Adults (15 years and older)	23,371	72,450	8,281	35.4	11.4	2.525	<0.001	(1.941–3.284)	1.50	0.003	(1.102–2.002)
Children (under 15)	315	977	56	17.8	5.7	1			1		
Marital status											
Married	18,072	56,023	6,320	35.0	11.3	1			1		
Not married	5,606	17,379	2,016	36.0	11.6	1.09	0.007	(1.008–1.122)	1.182	0.004	(1.013–1.347)
Employment status											
Employee	8,912	27,627	3,163	35.5	11.5	1.01	0.694	(0.965–1.055)			
Unemployed	14,764	45,768	5,170	35.0	11.3	1					
Type of HIV											
HIV type 1	23,534	72,955	8,285	35.2	11.4	0.906	0.514	(0.681–1.231)	NS	NS	
Other types (Type 2 or Type 1 + 2)	132	409	46	34.8	11.2	1					
Category of CD4 count in cells/μL											
>350	7,703	23,879	642	8.3	2.7	1			1		
200–350	7,766	24,075	928	11.9	3.9	1.27	<0.001	(1.133–1.384)	0.37	<0.001	(0.018–0.517)
100–200	4,168	12,921	3,638	87.3	28.2	16.51	<0.001	(15.133–17.806)	5.07	<0.001	(4.564–5.804)
<100	4,004	12,412	3,124	78.0	25.2	14.05	<0.001	(12.824–15.452)	5.92	<0.001	(5.091–6.543)
WHO Stage of HIV infection											
Early stage (I and II)	12,381	38,381	1,759	14.2	4.6	1			1		
Advanced stage (III and IV)	11,305	35,046	6,578	58.2	18.8	4.85	<0.001	(4.701–5.286)	3.66	<0.001	(3.045–3.975)
ART regimens											
TDF + 3TC + EFV	18,855	58,451	6,451	34.2	11.0	1.21	<0.001	(1.052–1.762)	NS	NS	
AZT + 3TC + NVP	2,284	7,080	1,195	52.3	16.9	1.18	<0.001	(1.087–2.123)			
Other ART regimes of first line	2,547	7,896	691	27.1	8.8	1					

$ means number of people in attrition.

## Discussion

In this study, we analyzed retention and attrition in the antiretroviral treatment program between September 2007 and April 2020 at nine large-volume care sites for people living with HIV in Guinea.

### Probability of Retention

After a median follow-up of 3.1 years, 35.2% were no longer retained in the program, for an overall retention rate of 64.8%. This probability was 86.0% at 6 months, 76.2% at 12 months and 70.2% at 24 months. The authors of a systematic review from studies in sub-Saharan Africa showed weighted average retention rates of 79.1%, 75.0%, and 61.6% at 6, 12, and 24 months, respectively [16].

The retention rate in our study is much lower than the proposed national goal (80% retention rate for children under 15 years of age after 12 months of follow-up and 90% retention rate for adults 15 years of age and older after 12 months of follow-up) [9]. Huge efforts are needed for the success of the ART program in Guinea. The focus could be on actively seeking out and investigating those lost to treatment to find out more about the reasons behind them, decentralizing and deconcentrating ART, and implementing innovative community-based strategies to improve access to care and retention of patients on ART.

### Attrition and its Components

The cumulative probability of attrition was 35.2%, or a cumulative incidence of 11.4 per 100 person-years. Death and loss to follow-up were the two components for attrition. Cumulative mortality at the date of data extraction was 6.8%. This death rate increased very gradually over the years of follow-up on ART, which is comparable to the observations made by other authors of studies conducted in low- and middle-income countries [17–19].

Although mortality is important, loss to follow-up was the main reason for attrition in our cohort. Of the 23,686 patients included in our cohort, 28.4% of patients were lost to follow-up by the end of the study. These results are comparable to those found elsewhere [20–24].

Several hypotheses can be put forward to explain the high number of people lost to follow-up. Some patients may change treatment sites to obtain ART from other sites without their original sites being informed. These patients may be categorized as lost to follow-up at their home sites, despite continuing to take ART elsewhere. Twaya et al. found in a Malawian cohort that 21% of patients considered lost to follow-up in their home sites were obtaining supplies from other sites [25]. Brinkoff et al. also reported in their 2009 meta-analysis that this “silent transfer to other treatment sites” is the main reason for non-return to the initial treatment site of patients on ART [22]. This hypothesis remains valid for the Guinean context, especially in Conakry, where the majority of HIV treatment sites are located. Patients may change treatment sites for many reasons including financial barriers in terms of transport costs or the cost of biological tests to be paid by the patient [22, 26, 27].

Poor quality of care may also be a reason for PLHIV to change treatment sites. Some services are not available in many sites (Drugs for opportunistic infections, materials for biological monitoring are not offered free of charge to PLHIV everywhere. Care is also not usually comprehensive. The nutritional management and food management are not integrated in most sites. This lack of continuity and comprehensiveness of care could be a significant reason for the loss of patients [9, 15].

### Predictors of Attrition

We found that the clinical, immunological, and virological status of the patient at the initiation of ART was the main predictor of attrition among patients living with HIV on antiretroviral treatment. Patients who initiated ART with a CD4 count <100 cells/μL were 5.92 times more prone to attrition than patients who initiated ART with a CD4 count > 350 cells/μL. The same is true for patients who started treatment at an advanced stage (III and IV of the WHO classification) of HIV infection, who were 3.66 times more at risk of attrition than patients who initiated ART at an early stage (stage I and II of the WHO classification). Several previous studies have reported similar results [27–33]. Patients with advanced HIV infection at the time of initiation of ART are subject to attrition due to high mortality and morbidity. There is evidence that initiation of ART at an advanced clinical and immunological stage is associated with high mortality [34].

It is therefore essential to mobilize resources for the reinforcement of laboratory equipment, the training of health workers, and the provision of health facilities with the health products necessary for the care of patients with advanced HIV infection.

This study also showed that patients who initiated ART during the period 2012–2015 were 2.81 times more at risk of attrition than patients who initiated ART between 2017 and 2020. This result could be explained by the impact of the Ebola virus disease epidemic on health systems in West Africa, including Guinea between 2014 and 2016. It should be recalled that the period 2014–2015, belonging to the period 2012–2015 was the period of high transmission of the Ebola virus in Guinea. This has led in Guinea, as in the affected countries of West Africa, to a drastic result in the use of health services, which can therefore lead to an increase in the rate of attrition among patients on ART. This hypothesis is supported by a study that has assessed the impact of the Ebola virus disease epidemic in Guinea and West Africa [35]. Another explanation for this result is linked to the significant change made in improving HIV care in Guinea during the period 2017–2020. It was during this period that the “Treat everyone” strategy has been strongly implemented. The “treat All” approach gives patients a strong incentive to start ART at an early stage of HIV infection; which is a factor improving patient survival. This was not the case between 2012 and 2015, when putting HIV patients on ART was selective (criterion according to the CD4 count - 200 and - 350 cells/μL).

Our study also found that patients who initiated ART as adults (15 years and older) were 1.50 times more at risk of attrition than patients who initiated ART young (less than 15 years). Our results are contrary to those of several studies conducted in Africa which found that young age was a risk factor for attrition [27, 33, 36, 37]. This discrepancy could be attributed to the difference in the study population.

In our study, unmarried patients were 1.18 times more at risk for attrition than married patients. A study conducted in Kenya in 2020 showed that unmarried status was associated with both loss to follow-up and mortality in the cohort of patients in an ART program [20]. This finding could be explained by the failure of unmarried HIV patients to disclose their HIV status to their sexual partners. This could lead to an increased likelihood of attrition or discontinuation of ART among these single patients [38]. For this purpose, we recommend the integration of support systems for unmarried patients through PLHIV support groups or improving disclosure to sexual partners.

### Strengths and Study Implications

Our study assessed the retention of people living with HIV in a treatment program implemented in several large cohort sites in Guinea and identified risk factors associated with attrition. It covered a long follow-up period (13 years) and included a large sample of patients on ART (over 23,000 patients). The cohort of patients followed in the sites involved in this study represented more than 45% of the national cohort of patients on ART in all 142 sites in the country.

It is also important to recall that since the advent of free ART in 2007, this is the first large-scale study that has not only estimated retention, but also analyzed the factors that influence attrition (i.e., loss to follow-up and death) of patients living with HIV in an ART program in Guinea.

### Limitations

Despite the strengths mentioned above, this study has some limitations. The data analyzed were collected retrospectively through longitudinal patient follow-up databases, which presents a risk of information bias due to under-reporting of some events of interest (i.e., deaths and transfers), and some key variables such as CD4.

Mortality estimation was based on reported deaths only. Adjustment for mortality was not made due to lack of information on deaths among those lost to follow-up. Data on potential predictors such as education, socio-economic level, weight, height, and hemoglobin level were missing. Also, the study did not investigate the reasons why patients are lost to follow-up and go to other care sites. Finally, the sample for this study was drawn from large-cohort sites that had more or less a good electronic management system for patients on ART, so small-cohort sites were absent. It should also be noted that the inclusion of only one site outside of Conakry, and the fact that all sites in the study are supported by NGO partners, limits the representativeness of this study for the entire country, Guinea. Also, the use of the availability of electronic databases as an inclusion criterion for ART centers could be a bias in this study, as retention or attrition could be worse or better in centers without an electronic database archive. However, this study provides a solid evidence base for the Conakry sites and for the Ministry of Health and National AIDS and Hepatitis Control Program authorities in improving retention in the ART program in Guinea. It also provides a good evidence base for further studies on this topic at the national level.

### Conclusion

This study showed a high attrition rate of HIV patients on ART, making the retention rate in our study much lower than the proposed national target of 90%. This suggests that mechanisms to improve retention of patients on ART for life should be put in place or strengthened: implementation of online electronic patient data management platforms, strengthening of community-based psychosocial support mechanisms for patients, strengthening of the implementation of innovative approaches to HIV management (3 or 6 months appointments, prevention of tuberculosis using Isoniazid in TB negative patients, etc.), and strengthening of nutritional and dietary support. Another study (mixed-methods study) with direct questioning of patients could also complete this research through an analysis of a larger number of predictors of attrition.
